# Prevalence and Associated Factors of Hypertension among Adults in Durame Town, Southern Ethiopia

**DOI:** 10.1371/journal.pone.0112790

**Published:** 2014-11-21

**Authors:** Tsegab Paulose Helelo, Yalemzewod Assefa Gelaw, Akilew Awoke Adane

**Affiliations:** 1 Zonal Health Department, Southern Nations Nationalities and People's Health Bureau, Durame, Ethiopia; 2 Department of Epidemiology and Biostatistics, Institute of Public Health, College of Medicine and Health Sciences, University of Gondar, Gondar, Ethiopia; Hospital de ClÃ­nicas de Porto Alegre, Brazil

## Abstract

**Background:**

To date, non-communicable diseases, such as cardiovascular diseases, are becoming severe public health challenges particularly in developing countries. Hypertension is a modifiable risk factor that contributes the leading role for mortality. The problem is significant in low- and middle-income countries like sub-Saharan Africa. However, there are limited studies in developing countries, particularly in Ethiopia. Hence, determining the magnitude of hypertension and identifying risk groups are important.

**Methods:**

A community based cross sectional study was conducted in April 2013 among adults (age>31 years) old. A systematic sampling technique was used to select a total of 518 study participants. Data were collected after full verbal informed consent was obtained from each participant. Multivariable logistic regressions were fitted to control the effect of confounding. Adjusted Odds ratios (OR) with their 95% confidence intervals (95% CI) were calculated to measure associations. Variables having P-value <0.05 were considered as significant.

**Results:**

The overall prevalence of hypertension in Durame town was 22.4% (95% CI: 18.8–26.0). Nearly 40% of hypertensive patients were newly screened. Male sex [AOR  = 2.03, 95% CI; 1.05–3.93], age [AOR  = 29.49, 95% CI; 10.60–81.27], salt use [AOR  = 6.55, 95% CI; 2.31–18.53], eating vegetable three or fewer days per week [AOR  = 2.3,95% CI; 1.17–4.51], not continuously walking at least for 10 minutes per day [AOR  = 7.82, 95% CI; 2.37–25.82], having family history of hypertension [AOR  = 2.46, 95%CI; 1.31–4.61] and being overweight/obese [AOR  = 15.7, 95% CI 7.89–31.21)] were found to be risk factors for hypertension.

**Conclusions:**

The prevalence of hypertension is found to be high. Older age, male sex, having family history of hypertension, physical inactivity, poor vegetable diet, additional salt consumption and obesity were important risk factors associated with hypertension among adults. Community level intervention measures with a particular emphasis on prevention by introducing lifestyle modifications are recommended.

## Introduction

The double burden of communicable and non communicable disease is an increasing public health challenge worldwide, especially in developing countries. World Health Organization (WHO) 2008 reported that non – communicable diseases contributed to 67% of the total deaths which occurred in low-and middle-income countries of which cardiovascular diseases were responsible for 48% of these deaths [1]. Hypertension is a global public health challenge due to its high prevalence and the concomitant risk of stroke and cardiovascular diseases in adults. It is estimated to cause 7.5 million deaths, about 12.8% of the total annual deaths in Sub – Saharan Africa [1]–[3].

Hypertension augments the risk of cardiovascular diseases, and it has been increasingly the risk of coronary heart disease, congestive heart failure, ischemic and hemorrhagic stroke, renal failure, and peripheral arterial diseases [4]. Treatment of hypertension and changing the life styles have been associated with a 40% reduction in the risk of stroke and about 15% reduction in the risk of myocardial infarction [1].

According to the 2010 global non-communicable disease status report, the prevalence of hypertension has been increasing over the past decades from 600 million in 1980 to nearly 1 billion in 2008 because of population growth and ageing [5].

From the previous studies, various risk factors have been associated with hypertension, including age, sex, obesity, physical activity, family history and socioeconomic status [1],[3],[5]. Reliable information about the prevalence and risk factors of hypertension is important for understanding the magnitude of the problem, identifying the risk groups and developing effective preventive strategies. Thus, the objective of this study was to determine the prevalence of hypertension and possible associated factors among adults of Durame Town, Southern Ethiopia.

### Ethical considerations

This study was carried out after getting ethical approval from the Institutional Review Committee of Institute of Public Health, University of Gondar. Before the ethical approval, the proposal was provided to reviewers to assure the ethical issues. Finally, the ethical review committee approved the oral consent by considering that the research has not serious harm to the study participants. Before the interview and measurements, the interviewer fully explained the purpose of the study to each participant and obtained full verbal informed consent from each study participant. To ensure confidentiality, names were not used in the questionnaire and reporting the results of the study. In addition, the collected information was locked with a key (hard copies) and password protected (soft copies). Participants found to be hypertensive during measurements were advised to visit the nearby health facility for further diagnosis and possible treatment.

## Methods and Materials

### Study design and study populations

A community based cross sectional study was conducted from April 1 to 30, 2013 in Durame town administration. Durame town is located 350 Kilo- meters south of Addis Ababa, the Ethiopian capital. The town administration has 3 *Kebeles* (the smallest administrative unit in Ethiopia) having 32,423 population according to 2007 Ethiopian Central Statistical Agency office report [6]. The study included adults whose age is >31 years and permanent resident (who lived in the area at least for six months) of Durame town.

### Sampling techniques and Sample Size determination

A systematic random sampling technique (i.e. every twelve households) was employed to select study participants. The first house hold was selected by lottery method and when there were more than one eligible adult in the household, only one was selected using lottery method. The required sample size of the study (536) was determined using single population proportion formula by considering: prevalence of hypertension 28.3% from previous study done in northwest Ethiopia [7], α = 0.05 (z = 1.96), the worst acceptable value from 24.3% to 32.3% (d = 0.04) and 10% possible non response rate.

### Data collection and analysis

Participants were interviewed using structured questionnaire consisted of demographic, behavioral, and clinical profiles [8]. The data collectors were clinical nurses supervised by two senior health officers. Training and practical demonstrations on the interview techniques and measurement procedures were given to data collectors for three consecutive days. Blood pressure measurements were obtained in the left arm with seated position using standard mercury sphygmomanometer BP cuff. Participants were inquired whether they had consumed any hot beverage, such as tea or coffee, smoked cigarette or undertaken any vigorous-intensity physical activity 30 minutes before measurement otherwise BP measurement was postponed for 30 minutes. The second BP measurement was taken after five minutes of the first measurement. Finally, the average of the two readings was considered as the final BP of each participant. Hypertension was defined as systolic BP ≥140 mmHg or diastolic BP ≥90 mmHg or reported use of regular anti-hypertensive medication(s). Height of the participants was measured at standing upright position with no shoe. Weight of participants was also measured while wearing light clothes using a calibrated weight scale. Based on the weight and height measurements of respondents, body mass index (BMI) was calculated (i.e. weight/height squared) and was classified as underweight (<18.5), normal (18.5–24.99), overweight (25–29.99) and obese (≥30).

Data were entered to computer using EPI INFO and transferred to SPSS version 20 for analysis. Both bivariable and multivariable logistic regression models were used to identify associated factors of hypertension. Variables having P-value ≤0.2 in the bivariable analysis were remained in the multivariable model to control the effect of confounders. The Hosmer -Lemeshow goodness-of- fit statistic was used to assess the fitness of the model. Odds ratios (OR) with their 95% confidence intervals (95% CI) were calculated to measure the strength of association. P value <0.05 was considered as significant.

## Results

### Socio-demographic and socio economic characteristics of respondents

Five hundred eighteen (with a response rate of 96.6%) participants were included in this study with the mean age of 47.4 (±12.2SD) years. Slightly more than half (55.8%) of them were females. More than three fourth were married (76.6%) and about a third of them were housewives (31.7%) [Table 1].

**Table 1:** Socio-demographic characteristics of respondents in Durame town administration, Southern Ethiopia, April 2013 (n = 518).

Characteristics		Frequency	%
Sex	Male	229	44.2
	Female	289	55.8
Age	31–40	210	40.5
	41–50	114	22
	>50	194	37.5
Ethnicity	Kembata	405	78.2
	Tembaro	17	3.3
	Hadya	32	6.2
	Alaba	18	3.5
	Walayta	15	2.9
	Amhara	25	4.8
	Others**	6	1.2
Religion	Protestant	349	67.4
	Orthodox	66	12.7
	Catholic	88	17
	Muslim	15	2.9
Marital status	Single	55	10.6
	Married	397	76.6
	Divorced	17	3.3
	Widowed	49	9.5
Educational level	No formal education	134	25.9
	primary education(1–8)	113	21.8
	secondary education(9–12)	126	24.3
	Diploma and above	145	28
Occupation	Government Employee	128	24.7
	Daily Laborer	23	4.4
	Merchant	86	16.6
	House Wife	164	31.7
	Retired	35	6.8
	Farmer	66	12.7
	Others*	16	3.1
monthly income	<1214	336	64.9
	= >1214	182	35.1

* = 10 NGO Employee and 6 driver.

** = 4 Dongaw and 2 Sidama.

### Prevalence of hypertension

The mean systolic and diastolic BP readings were 120 (±15.3 SD) and 78(±10.1 SD) mmHg, respectively. The overall prevalence of hypertension was 22.4% (*95% CI*: 18.8–26.0). The prevalence of hypertension was slightly higher in males than females (X^2^ = 3.54, p – value 0.045). Among hypertensive cases, more than one thirds (39.6%) of them were newly screened - who did not know that they had hypertension.

### Factors Associated with Hypertension

Among modifiable risk factors assessed in this study; physical inactivity, vegetable eating habit, and use of top added salt on plate were significantly associated with hypertension. If participants of the study use top added salt on plate, then they were [AOR  = 6.55, 95%CI; 2.31–18.53] more likely to be hypertensive than their counter parts. In this study, the prevalence of hypertension was higher in older ages. For instance, those who were in 40–50 years category had AOR of 8.88(95% CI: 2.92–27.04) as compared to those 31–40 years old. Participants who did not walk at least for 10 minutes continuously on daily basis were about eight times [AOR  = 7.82,95% CI; 2.37–25.82] more likely to be hypertensive. Whereas, adults who did not eat vegetables for more than three days on their weekly menu were about two times [AOR  = 2.30, 95% CI; 1.17–4.51] high likely to be hypertensive than those eat daily. Participants who had family history of hypertension in this study were also found to be significantly at higher risk of hypertension [AOR  = 2.46, 95%CI; 1.31–4.61].

Nearly three fourth of participants (72%) had a normal BMI, whereas the rest were either overweight or obese. Overweight/obesity was found to be strong risk factor for hypertension [AOR  = 15.7, 95% CI; 7.89–31.21] (Table 2).

**Table 2:** Multivariate analysis of factors associated with hypertension, Durame Town Administration, Southern Ethiopia, April 2012(n = 518).

Variable		Hypertension		COR(95%CI)	AOR(95%CI)
		Yes (%)	No (%)		
Sex	Male	60 (26.2%)	169 (73.8%)	1.48 (.98–2.24)	2.03(1.05–3.93)
	Female	56 (19.4%)	233 (80.6%)	1	1
Age	31–40 Years	6 (2.9%)	204 (97.1%)	1	1
	41–50 Years	10 (15.8%)	96(84.2%)	6.37(2.45–16.57)	8.88(2.92–27.04)
	>50 Years	102(52.6%)	92(47.4%)	30.67(12.98–72.42)	29.49(10.70–81.27)
vegetable eating habit/week	3 or fewer days	87(27.8%)	226(72.2%)	2.34(1.47–3.72)	2.30(1.17–4.50)
	4–7 days	29(14.1%)	176(85.9%)	1	1
Salt use	Yes	17(47.2%)	19(52.8%)	3.46(1.74–6.91)	6.54(2.3–18.53)
	No	99(20.5%)	383(79.5%)	1	1
number of days walking 10 min/week	None in a week	22(71.0%)	9(29.0%)	13.55(5.87–31.28)	7.82(2.37–25.82)
	1–3 day	48(25.8%)	138(74.2%)	1.93 (1.22–3.02)	1.48(.74–2.94)
	4–7 day	46(15.3%)	255(84.7%)	1	1
Family history of HTN	Yes	71(33.3%)	142(66.7%)	2.89(1.89–4.42)	2.46(1.30–4.61)
	No	45(14.8%)	260(85.2%)	1	1
BMI	Normal	42(11.3%)	330(88.7%)	1	1
	Under Weight	1(5.0%)	19(95.0%)	0.41(0.05–3.17)	0.236(0.26–2.14)
	Overweight/obese	73(57.9%)	53(42.1%)	10.82(6.71–17.45)	15.7(7.89–31.21)

## Discussion

Hypertension is the leading risk factor for cardiovascular diseases in sub-Saharan African countries [9]. In this study, roughly one in five adults (31 years and above) remarkably had hypertension (22.4%). This result is slightly higher than a community based cross - sectional studies done in sub- Saharan Africa countries; Sidama Zone, Ethiopia (18.8%), Eritrea (10.3%) and Nigeria (16%) [10]–[12]. This discrepancy could be explained in two ways; this study is considered only urban setting whereas the former studies included urban and rural settings. And the other reason for the discrepancy might be the age difference in the study population (≥31 years of age with 45.8±11.7 mean ages in our case were included while other studies included adult population aged 15–90 year). However, it is lower than the findings from developed countries such as United States and Portugal [17],[22].

In this study, the prevalence hypertension was considerably higher in males (26.2%) than females (19.4%). This is also in line with previous studies done in Ethiopia [7],[15],[16] and in low and middle income countries [16]–[18]. As many studies reported, the prevalence of hypertension has a positive association with age [7],[10]–[14],[19]. This is due to the fact that the biological effect of increased arterial resistance due to arterial thickening as one gets older [3].

Almost one quarter of adults was overweight/obese (24.2%) in this study; and 58% of them were hypertensive which was consistent with findings reported in community based studies in sub – Saharan Africa countries [7],[10]–[16],[18].

Likewise the previous studies [7],[10],[15],[16], family history of hypertension was significantly associated with the occurrence of hypertension. This is due to the fact that family members may share similar life style and genetic factors. It has been demonstrated that people who reported ever use of top added salt on plate had a positive association with hypertension which was evidenced elsewhere [20],[21]. It is already an established fact that a high salt diet disrupts the natural sodium balance in cells. It causes fluid to stay longer which increases the pressure exerted by the blood on arteries resulting in high blood pressure [3].

Previous studies [7],[10],[12],[15],[16], suggested that self-reported history of diabetes, alcohol consumption and cigarette smoking were significantly associated with hypertension. Paradoxically, in this study, self-reported history of diabetes, alcohol consumption and cigarette smoking have no significant relationship with hypertension. The inconsistency of these findings could be owing to low prevalence of these risk factors in the general community and particularly in females.

The potential limitations worth of the study were: firstly this study did not include the rural dwellers. Secondly, most of the respondents did not know their exact birth date and this may under or over estimate the prevalence of hypertension since there is no reliable measurement of age. Blood pressure measurements were taken on a single day. Additionally, it's limited to only behavioral and physical measurements of the participants that did not include biochemical measurements.

## Conclusion

The prevalence of hypertension was found to be high among adults (age ≥31) in Durame town. Factors like, family history of hypertension, physical inactivity, age, being obese/overweight, dietary habits and use of excess salt on plate were found to be significantly associated with hypertension. We recommend that the policy makers need to focus on community level intervention through integration with the open door health extension program. It is also better to give special emphasis for health education regarding the daily live events like healthy dietary habit and regular exercise.
